# Supplementary material for: Pixel super-resolved virtual staining of label-free tissue using diffusion models
Source: Nat Commun. 2025 May 30;16:5016. doi: 10.1038/s41467-025-60387-z (PMC12125245; doi:10.1038/s41467-025-60387-z)
Supplement: Supplementary file 1 — Supplementary Information [file 41467_2025_60387_MOESM1_ESM.pdf]

Supplementary Information for

Pixel super-resolved virtual staining of label-free tissue using diffusion models

Yijie Zhang[†,1,2,3], Luzhe Huang[†,1,2,3], Nir Pillar[1,2,3], Yuzhu Li[1,2,3], Hanlong Chen[1,2,3], and Aydogan Ozcan[*,1,2,3,4]

[1]Electrical and Computer Engineering Department, University of California, Los Angeles, CA, 90095, USA.

[2]Bioengineering Department, University of California, Los Angeles, CA, 90095, USA.

[3]California NanoSystems Institute (CNSI), University of California, Los Angeles, CA, 90095, USA.

[4]Department of Surgery, University of California, Los Angeles, CA, 90095, USA.

*Correspondence: Aydogan Ozcan, ozcan@ucla.edu

[†] Equal contributing authors

Supplementary Note 1: Brownian bridge diffusion process

Given a target image $x_0 \in \mathbb{R}^{H\times W\times C}$ and a conditional image $y \in \mathbb{R}^{H\times W\times C}$, the forward process of Brownian Bridge diffusion model[1] with a total sampling step T can be defined as:

$$q(x_t|x_0, x_T = y) = \mathcal{N}\big((1-m_t)x_0 + m_t y, \delta_t I\big) \tag{1}$$

where

$$m_t = \frac{t}{T} \qquad \delta_t = \frac{2t(T-t)}{T^2}$$

Given the transition probability $q(x_t|x_0, x_T = y)$, the intermediate states can be computed as:

$$x_t = x_0 + m_t(y - x_0) + \sqrt{\delta_t}\epsilon_t \tag{2}$$

$$x_{t-1} = x_0 + m_{t-1}(y - x_0) + \sqrt{\delta_{t-1}}\epsilon_{t-1} \tag{3}$$

where $\epsilon_t,\ \epsilon_{t-1} \sim N(0, I)$. By removing x_0 in Eq. (2) and Eq. (3), the transition probability $q(x_t|x_{t-1}, y)$ can be derived as:

$$q(x_t|x_{t-1}, y) = \mathcal{N}\left(x_t;\ \frac{(1-m_t)}{(1-m_{t-1})}x_{t-1} + \left(m_t - \frac{(1-m_t)}{(1-m_{t-1})}m_{t-1}\right)y, \delta_{t|t-1}I\right) \tag{4}$$

where $\delta_{t|t-1}$ is calculated as:

$$\delta_{t|t-1} = \delta_t - \delta_{t-1}\frac{(1-m_t)^2}{(1-m_{t-1})^2} \tag{5}$$

In the reverse process, the diffusion process starts from the conditional image $y \in \mathbb{R}^{H\times W\times C}$ by setting the input $x_T = y$. Given the input x_T, the x_{t-1} can be predicted by:

$$p_\theta(x_{t-1}|x_t, y) = \mathcal{N}\big(x_{t-1}; \mu_t'(x_t, y), \tilde{\delta}_t I\big) \tag{6}$$

where $\mu_t'(x_t, y)$ is the predicted mean value of the noise, and $\tilde{\delta}_t$ is the variance of noise at each step.

The training objective for the Brownian Bridge diffusion process is based on optimizing an Evidence Lower Bound (ELBO)[2], which can be denoted as:

$$ELBO = -\mathbb{E}_q\big(D_{KL}\big((q(x_T|x_0, y)||p(x_T|y)\big) + \sum_{2}^{T} D_{KL}(q(x_{t-1}|x_t, x_0, y)||p_\theta(x_{t-1}|x_t, y)) - \log p_\theta(x_0|x_1, y)\big) \quad (7)$$

where $q(x_{t-1}|x_t, x_0, y)$ in the second term can be derived through Bayes' theorem and the Markov chain property:

$$q(x_{t-1}|x_t, x_0, y) = \frac{q(x_t|x_{t-1}, y)q(x_{t-1}|x_0, y)}{q(x_t|x_0, y)} \quad (8)$$

By comparing with Eq. (1) and Eq. (4), Eq. (8) can be derived as:

$$q(x_{t-1}|x_t, x_0, y) = \mathcal{N}\big(x_{t-1}; \tilde{\mu}_t(x_t, x_0, y), \tilde{\delta}_t I\big) \quad (9)$$

where the mean term $\tilde{\mu}_t(x_t, x_0, y)$ and the variance term $\tilde{\delta}_t$ are given by:

$$\tilde{\mu}_t(x_t, x_0, y) = \frac{\delta_{t-1}}{\delta_t}\frac{(1-m_t)}{(1-m_{t-1})}x_t + \left(1 - m_{t-1}\frac{\delta_{t|t-1}}{\delta_t}\right)x_0 + \left(m_{t-1} - m_t\frac{(1-m_t)}{(1-m_{t-1})}\frac{\delta_{t-1}}{\delta_t}y\right) \quad (10)$$

$$\tilde{\delta}_t = \frac{\delta_{t|t-1} \cdot \delta_{t-1}}{\delta_t} \quad (11)$$

By combining Eq. (1) and Eq. (10), $\tilde{\mu}_t(x_t, x_0, y)$ can be reformulated as:

$$\tilde{\mu}_t(x_t, x_0, y) = c_{xt}x_t + c_{yt}y - c_{\epsilon t}\big(m_t(y - x_0) + \sqrt{\delta_t}\epsilon_t\big) \quad (12)$$

where $c_{xt}, c_{yt,}\ c_{\epsilon t}$ are defined as:

$$c_{xt} = \frac{\delta_{t-1}}{\delta_t}\frac{(1-m_t)}{(1-m_{t-1})} + \frac{\delta_{t|t-1}}{\delta_t}(1 - m_{t-1}) \quad (13)$$

$$c_{yt} = m_{t-1} - m_t\frac{(1-m_t)}{(1-m_{t-1})}\frac{\delta_{t-1}}{\delta_t} \quad (14)$$

$$c_{\epsilon t} = (1 - m_{t-1})\frac{\delta_{t|t-1}}{\delta_t} \quad (15)$$

During training, the neural network ϵ_θ is trained to estimate the noise term $m_t(y - x_0) + \sqrt{\delta_t}\epsilon_t$ in Eq. (12), i.e.,

$$\mu_t'(x_t, y) = c_{xt} x_t + c_{yt} y - c_{\epsilon t} \epsilon_\theta(x_t, t) \tag{16}$$

Thus, the training objective Eq. (7) can be simplified to optimizing the loss between the sampled noise in the forward process and the estimated noise by the neural network:

$$L = \Sigma_t \gamma_t \mathrm{E}_{(x_0, y), \epsilon_t} \left\| m_t(y - x_0) + \sqrt{\delta_t} \epsilon_t - \epsilon_\theta(x_t, t) \right\|_2^2 \tag{17}$$

where γ_t is the weight for each t.

Supplementary Figures:

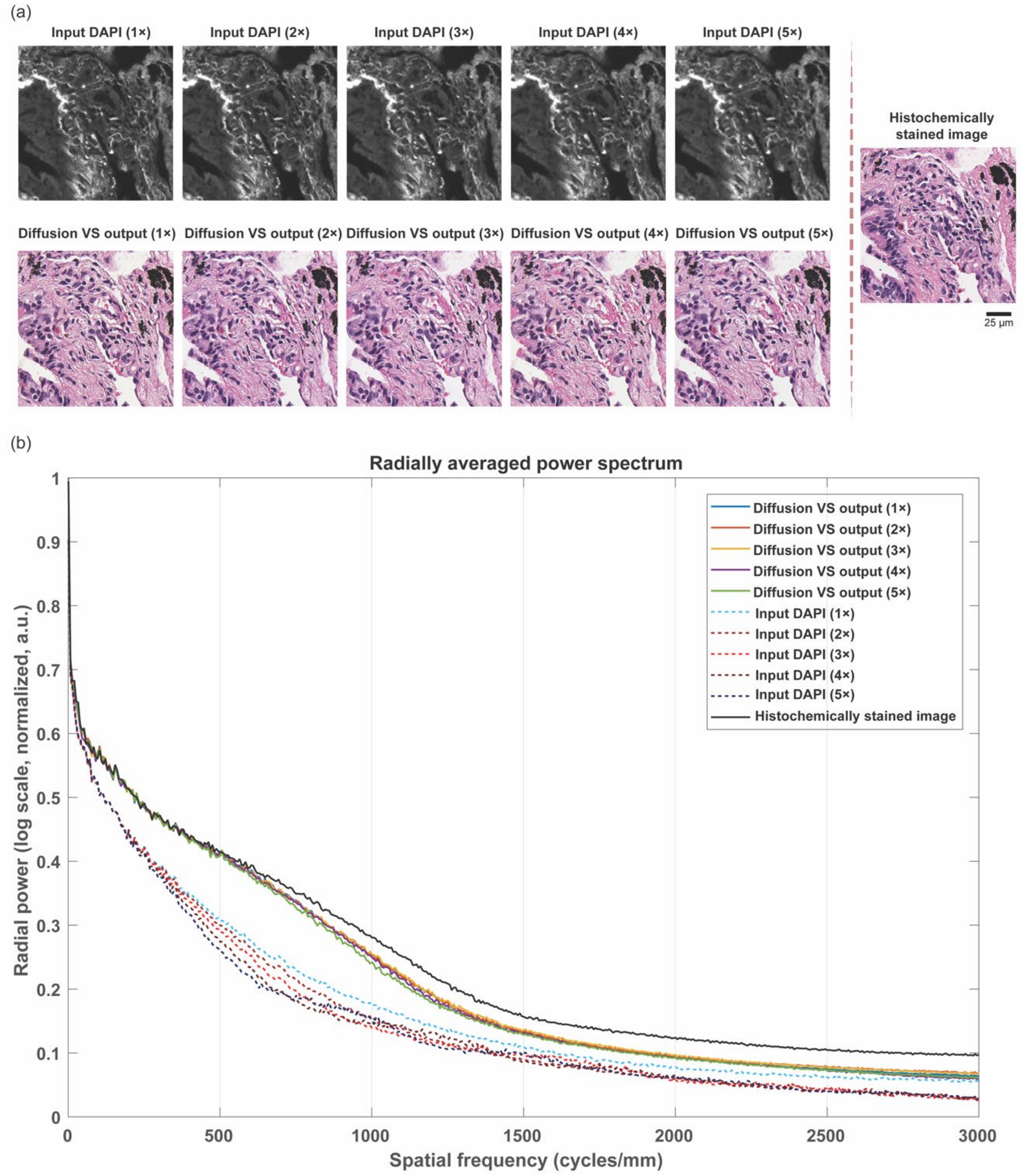

Supplementary Figure 1. Spatial frequency spectrum analysis of virtually stained images generated by diffusion-based VS models. (a) Input autofluorescence DAPI images, the virtually stained images generated by the diffusion models for different pixel super-resolution factors. The corresponding histochemically stained image is also displayed as ground truth. (b) The radially averaged power spectrum cross-sections corresponding to the images in (a).

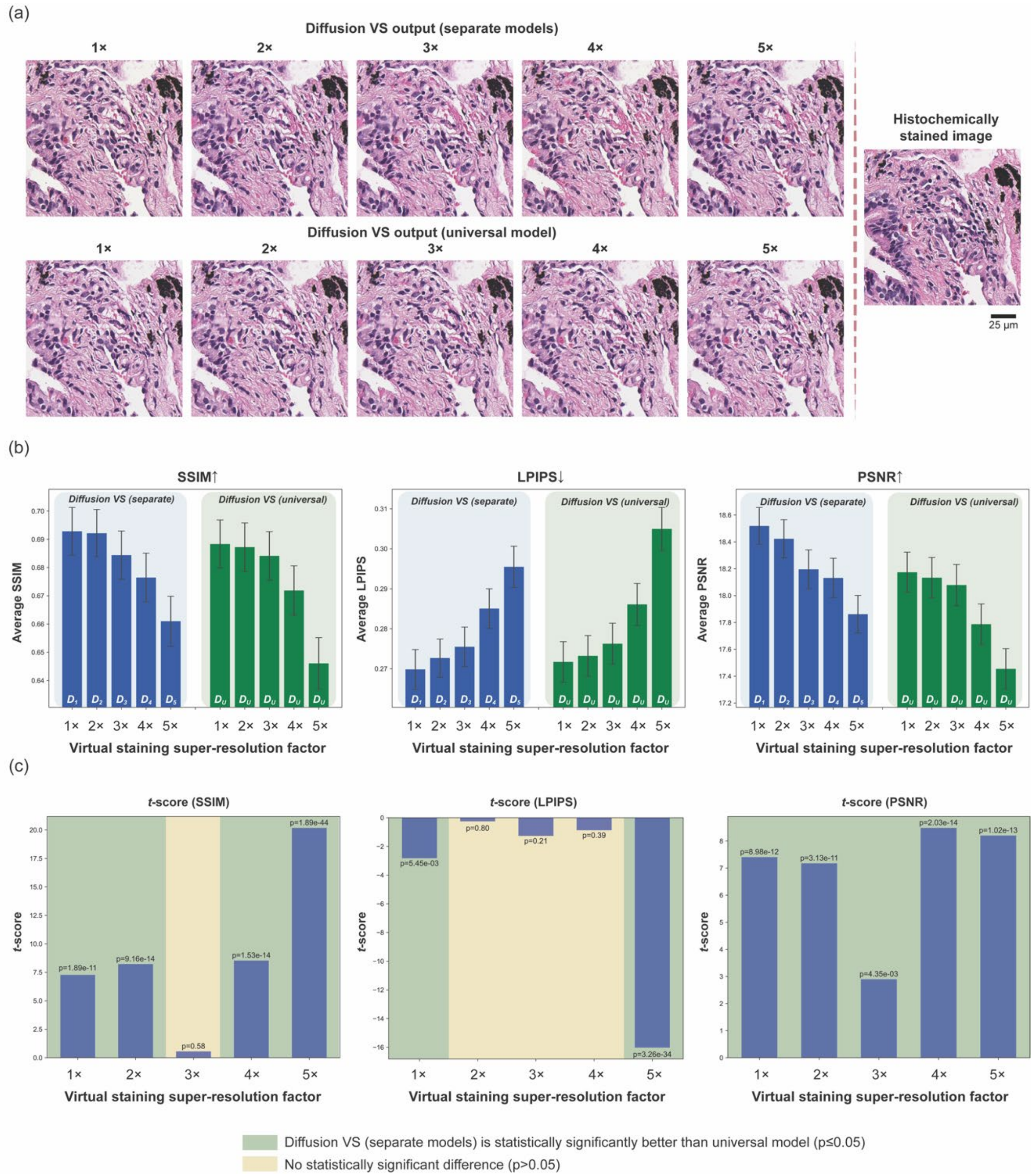

Supplementary Figure 2. Comparison of super-resolution virtual staining performances of dedicated and universal diffusion-based VS models. (a) Visual comparisons of virtually stained H&E images generated by separate dedicated models (top) and the universal model (bottom), utilizing the mean diffusion sampling strategy. The dedicated diffusion models were trained and tested individually for a particular spatial downsampling factor, whereas the universal model was simultaneously trained and tested across all pixel super-resolution factors (from 1× to 5×). All models were evaluated using autofluorescence images on n = 180 unique FOVs from 15 unlabeled lung samples. (b) Bar plots presenting the SSIM, LPIPS, and PSNR metrics, averaged across testing virtually stained images for the dedicated and the universal models. Error

bars represent the standard error of the mean. D_x represents the dedicated diffusion-based VS model for a particular super-resolution factor x, while D_U denotes the universal diffusion-based VS model. (c) Bar plots of t-scores comparing the performances of the dedicated models and the universal model for the same super-resolution factors. Green regions highlight statistically significant improvements in the virtual staining performance achieved by the dedicated models over the universal model. Source data are provided as a Source Data file.

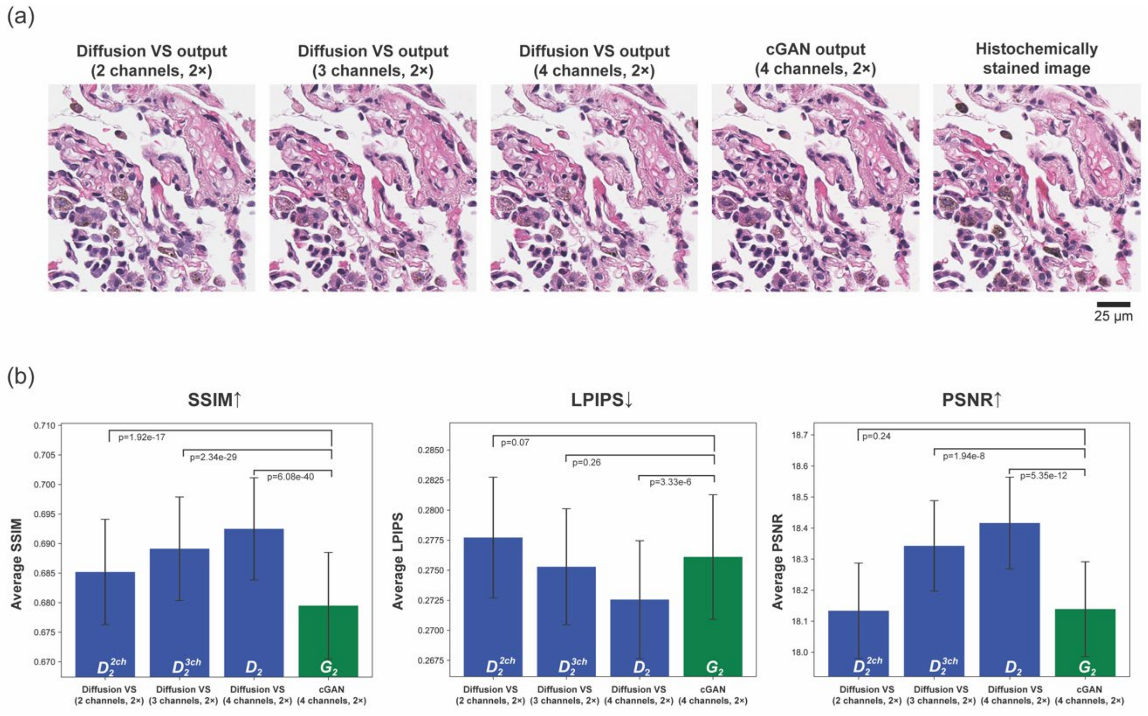

Supplementary Figure 3. Evaluation of super-resolution virtual staining performances of diffusion-based VS models using a reduced number of input autofluorescence channels. (a) Visual comparisons of virtually stained H&E images generated by diffusion-based VS models trained using 2 (DAPI and TxRed), 3 (DAPI, TxRed, Cy5), and 4 (DAPI, TxRed, FITC, Cy5) autofluorescence channels, with a 2× super-resolution factor. For reference, the corresponding virtually stained images generated by the cGAN model and the histochemically stained ground truth are also shown. (b) Bar plots illustrating the SSIM, LPIPS, and PSNR metrics averaged across testing virtually stained images for diffusion-based and cGAN-based VS models. These metrics were computed by comparing n = 180 sampled virtually stained images to histochemically stained H&E images from 15 blind testing lung samples. Error bars represent the standard error of the mean. The *p*-values resulting from paired *t*-tests comparing the diffusion VS models to the cGAN model are also provided. Source data are provided as a Source Data file.

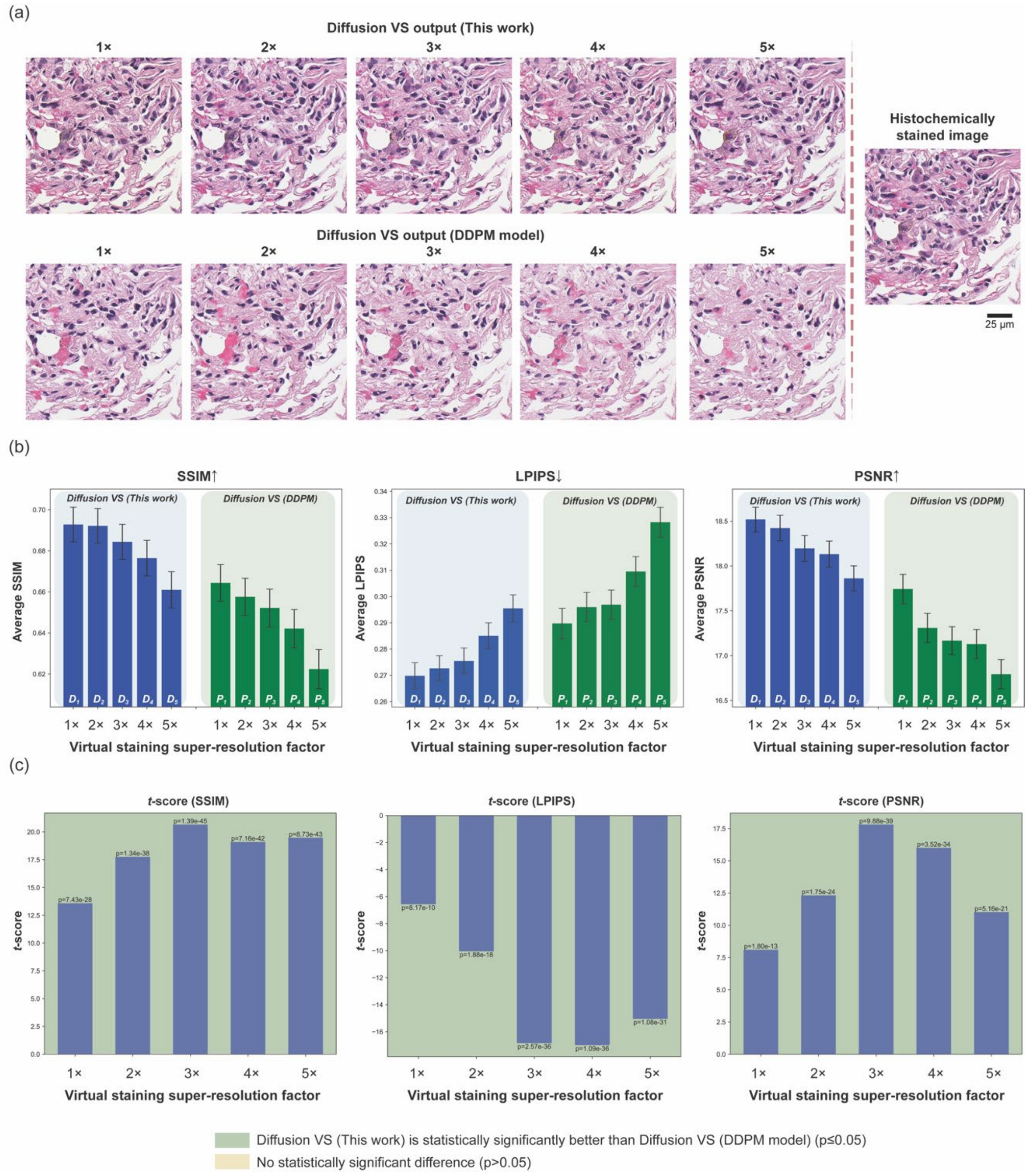

Supplementary Figure 4. Comparative evaluation of pixel super-resolution virtual staining performance of our diffusion VS models against DDPM-based models. (a) Visual comparisons of virtually stained H&E images generated by our diffusion VS models (top row) and the DDPM-based diffusion VS models (bottom row). Each model was independently trained and evaluated for specific super-resolution factors ranging from 1× to 5×. Evaluations were conducted using autofluorescence images from 180 distinct FOVs obtained from 15 unlabeled lung samples. (b) Bar plots illustrating quantitative comparisons of SSIM, LPIPS, and PSNR metrics, averaged across n = 180 test images virtually stained by our diffusion VS models and the DDPM-based VS models. Error bars indicate the standard error of the mean. Labels D_x and P_x

represent our VS models and DDPM-based VS models, respectively, for each super-resolution factor *x*. (c) Bar plots depicting *t*-scores comparing the performance differences between our VS models and the DDPM-based VS models at identical super-resolution factors. Green shaded areas highlight statistically significant improvements in virtual staining performance achieved by our VS models relative to the DDPM-based VS models. Source data are provided as a Source Data file.

Supplementary References

1. Li, B., Xue, K., Liu, B. & Lai, Y.-K. Bbdm: Image-to-image translation with brownian bridge diffusion models. in *Proceedings of the IEEE/CVF conference on computer vision and pattern Recognition* 1952–1961 (2023).
2. Ho, J., Jain, A. & Abbeel, P. Denoising diffusion probabilistic models. *Adv. Neural Inf. Process. Syst.* **33**, 6840–6851 (2020).
