# Supplementary figures and images for: Pixel super-resolved virtual staining of label-free tissue using diffusion models
Source: Nat Commun. 2025 May 30;16:5016. doi: 10.1038/s41467-025-60387-z (PMC12125245; doi:10.1038/s41467-025-60387-z)

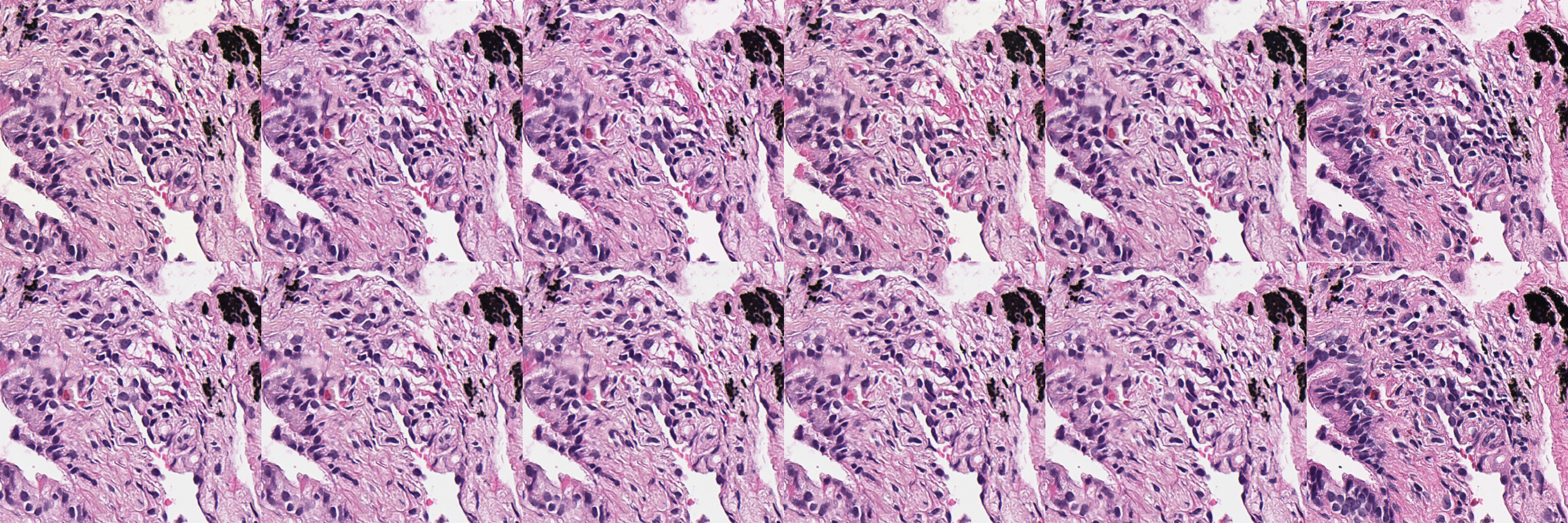

Supplement: Supplementary file 4 — Source Data [file 41467_2025_60387_MOESM4_ESM.tif]

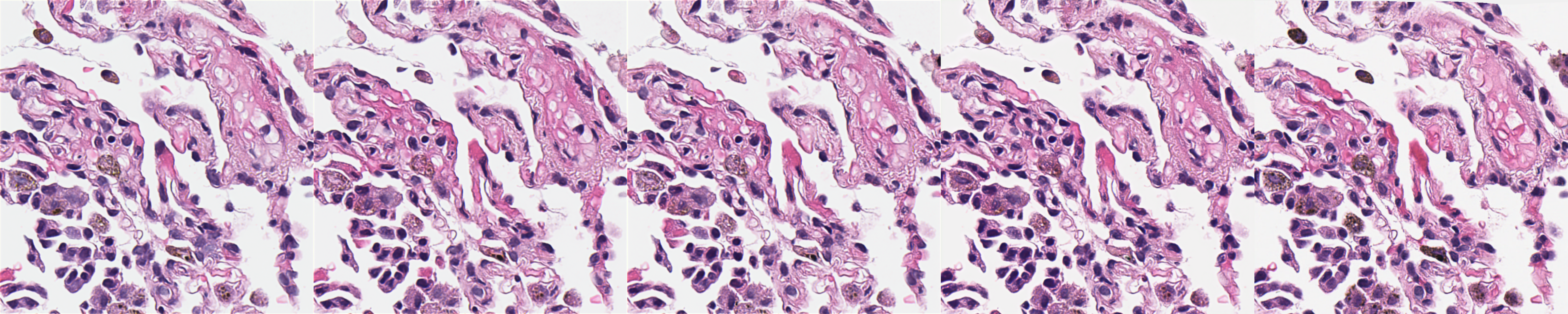

Supplement: Supplementary file 5 — Source Data [file 41467_2025_60387_MOESM5_ESM.tif]

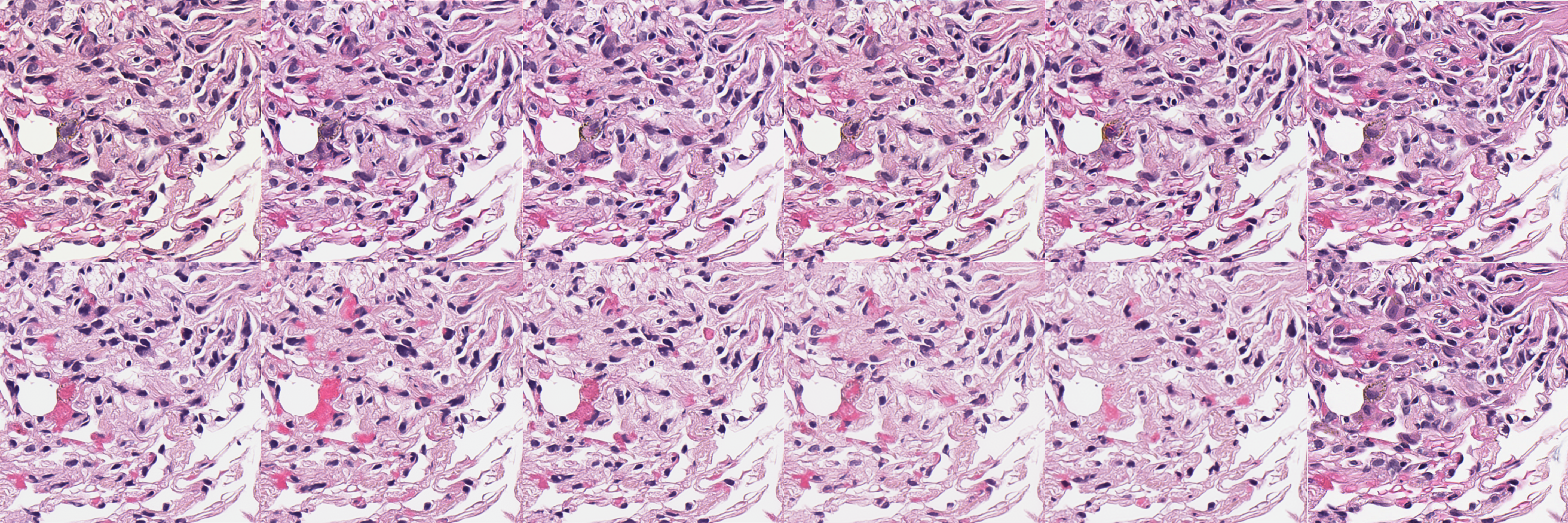

Supplement: Supplementary file 6 — Source Data [file 41467_2025_60387_MOESM6_ESM.tif]

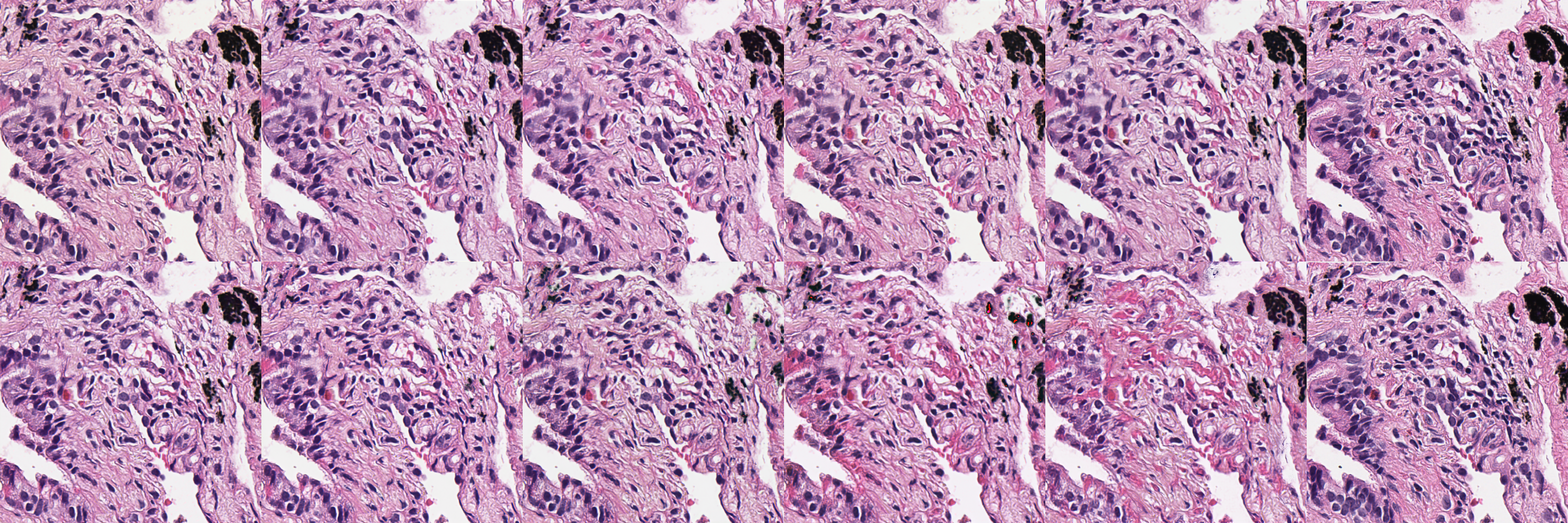

Supplement: Supplementary file 7 — Source Data [file 41467_2025_60387_MOESM7_ESM.tif]

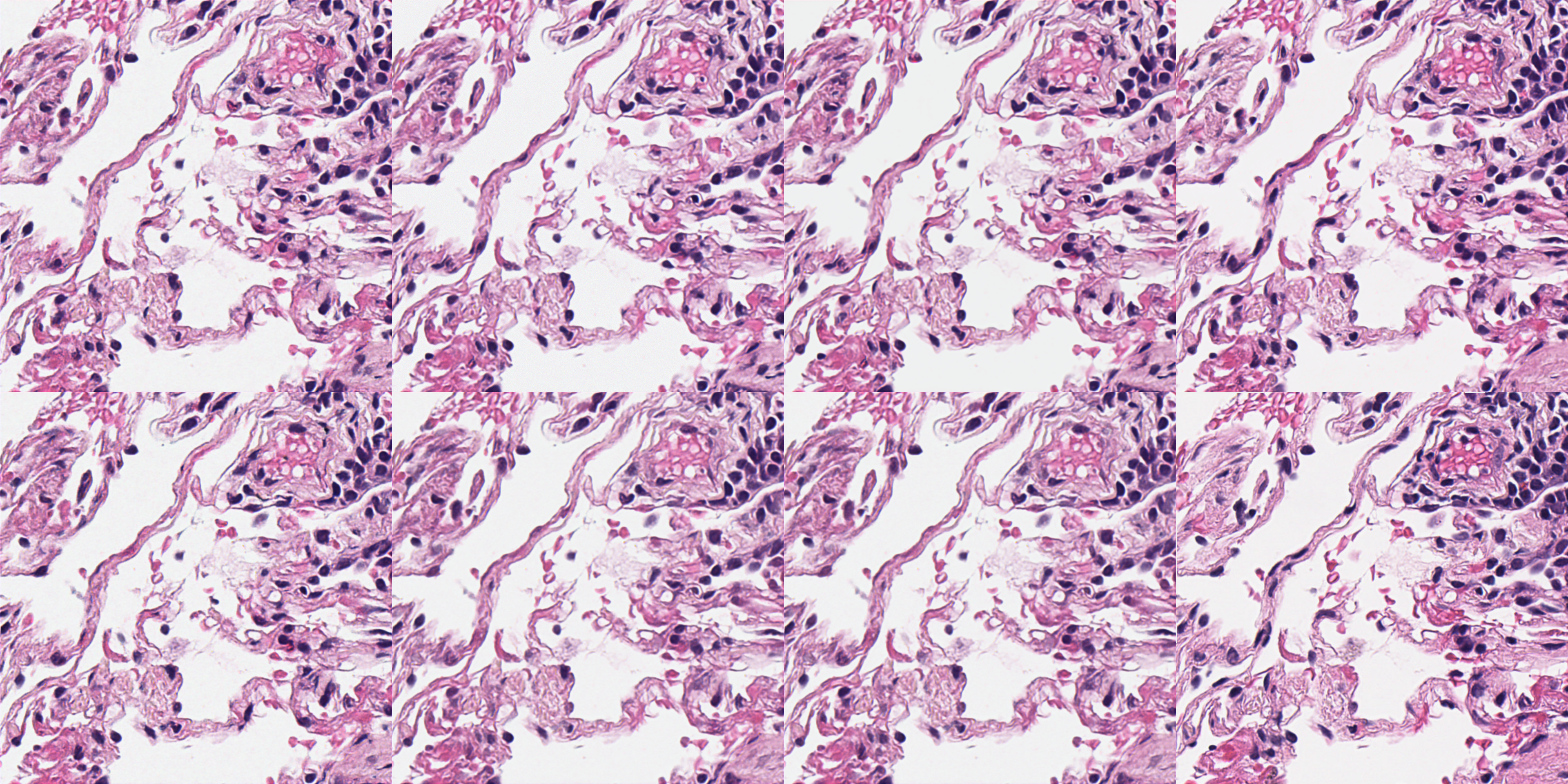

Supplement: Supplementary file 8 — Source Data [file 41467_2025_60387_MOESM8_ESM.tif]

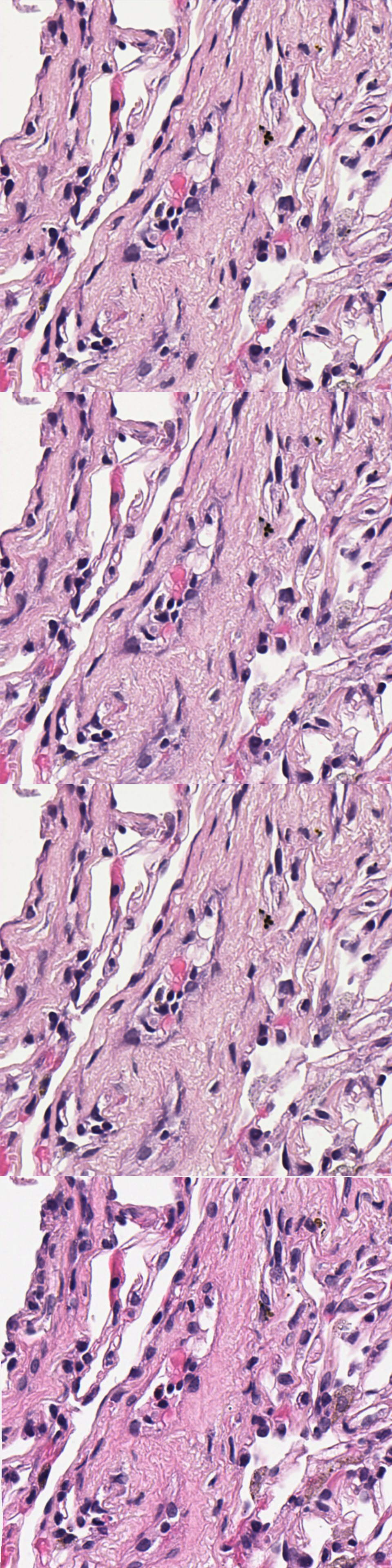

Supplement: Supplementary file 9 — Source Data [file 41467_2025_60387_MOESM9_ESM.tif]
